# Advantages of critical care ultrasound in primary survey: the experience of a medium size Emergency Department

**DOI:** 10.1186/2036-7902-6-S1-A27

**Published:** 2014-01-31

**Authors:** M Zanatta, V Cianci

**Affiliations:** 1 Emergency Department of Arzignano Hospital, ULSS 5 Ovest Vicentino, Vicenza, Italy

## Objective

Which is the real impact of critical ultrasound (CCUS) for an emergency physician (EP)?

## Patients and methods

We performed a retrospective analysis on 272 consecutive CCUS made during the primary survey (PS) in our medium size Emergency Department (ED).

We compared PS with discharge diagnosis and results were divided into 4 categories according to the role of CCUS: crucial for diagnosis (A), support clinical data (B), ruled out diagnosis (C), misleading (D).

## Results

In 23.22% of cases CCUS were performed for dyspnea, 16.85% for thoracic pain, 29.58% for abdominal pain, 13.85% for trauma, 9.36% were CUS, 4 in cardiac arrest settings and 10 to guide invasive procedures.

CCUS was crucial in 23.97% of patients, supported clinical data in 41.57% of cases, ruled out diagnosis in 32.58% of subjects, was misleading in 1.87% of cases. The correspondence between PS and discharge diagnosis (A+B) achieved 65.54%.

A definitive diagnosis was made in 83.87% of dyspnea, in 76% of CUS, in 64.55% of abdominal pain and in 53.33% of thoracic pain. Splitting patients with abdominal pain between those with more and less specific symptoms, the percentages were 100% and 22.22% respectively.

EFAST was never misleading and all complications were confirmed by a radiologist. All invasive procedures were successful and without complications. In cardiac arrest settings CCUS gave the indication to thrombolysis in two cases and to stop RCP in one subject.

In 36.9% of patients radiology examination weren't performed reducing diagnostic time and medical cost.

## Conclusion

Our experience showed that diagnostic capability was higher in lung ultrasound, EFAST and CUS respectively. It was lower in abdominal pain because symptoms were often functional disorders. Echocardiography had the lower percentage since it is the most difficult technique and cardiologist evaluation remains often mandatory.

In conclusion CCUS is an important instrument for the EP to save time and money. In particular lung ultrasound should be considered a necessary competence of the EP for the management of respiratory distress syndrome.

